# Supplementary material for: Differential Epigenetic Marks Are Associated with Apospory Expressivity in Diploid Hybrids of Paspalum rufum
Source: Plants (Basel). 2021 Apr 17;10(4):793. doi: 10.3390/plants10040793 (PMC8072704; doi:10.3390/plants10040793)
Supplement: Supplementary file 1 [file plants-10-00793-s001.zip › TableS1-S6-S7-S8.docx]

**Table S1:** Illumina sequencing specifications and results describing read counts for each library.

| Sample type | Context | Sample ID | Barcode | Sequencing Library_ID | Sample name | Total | Retained | Sum | Mean |
| --- | --- | --- | --- | --- | --- | --- | --- | --- | --- |
| Premeiosis  Highly Sexual | CG | #9_pre_P25 | AACACAA | aci_P25 | pre0_1 | 6361196 | 5934404 | 13887293 | 4629098 |
|  |  | #9_pre_P26 | AACGTGA | aci_P26 | pre0_2 | 4964215 | 4533832 |  |  |
|  |  | #12_pre_P27 | AAGGAAT | aci_P27 | pre0_3 | 3630091 | 3419057 |  |  |
|  | CHG | #9_pre_P1 | GCTACAA | pstI_P1 | pre0_1 | 3745558 | 3437449 | 10750386 | 3583462 |
|  |  | #9_pre_P2 | AGCATCA | pstI_P2 | pre0_2 | 5871210 | 4156221 |  |  |
|  |  | #12_pre_P3 | CCATCCA | pstI_P3 | pre0_3 | 3552859 | 3156716 |  |  |
|  | CHH | #9_pre_P31 | CACGCTA | eco_P31 | pre0_1 | 5940535 | 5092887 | 12737365 | 4245788 |
|  |  | #9_pre_P32 | CAGGGCA | eco_P32 | pre0_2 | 4728398 | 4127892 |  |  |
|  |  | #12_pre_P33 | CAGGTGT | eco_P33 | pre0_3 | 4541386 | 3516586 |  |  |
| **Total Premeiosis**  **Highly Sexual** |  |  |  |  |  | **43335448** | **37375044** |  | **4152782** |
| Premeiosis  Highly Aposporic | CG | #15_pre_P25 | AACACAA | aci_P25 | pre25_1 | 5379651 | 5189137 | 14233379 | 4744460 |
|  |  | #39_pre_P26 | AACGTGA | aci_P26 | pre25_2 | 4467226 | 4346611 |  |  |
|  |  | #39_pre_P27 | AAGGAAT | aci_P27 | pre25_3 | 4787147 | 4697631 |  |  |
|  | CHG | #15_pre_P1 | GCTACAA | pstI_P1 | pre25_1 | 3899249 | 3236767 | 7846191 | 2615397 |
|  |  | #39_pre_P2 | AGCATCA | pstI_P2 | pre25_2 | 2843163 | 2350699 |  |  |
|  |  | #39_pre_P3 | CCATCCA | pstI_P3 | pre25_3 | 2747355 | 2258725 |  |  |
|  | CHH | #15_pre_P31 | CACGCTA | eco_P31 | pre25_1 | 5931277 | 4862083 | 14729307 | 4909769 |
|  |  | #39_pre_P32 | CAGGGCA | eco_P32 | pre25_2 | 5003748 | 4147607 |  |  |
|  |  | #39_pre_P33 | CAGGTGT | eco_P33 | pre25_3 | 6860549 | 5719617 |  |  |
| **Total Premeiosis**  **Highly Aposporic** |  |  |  |  |  | **41919365** | **36808877** |  | **4089875** |
| Postmeiosis  Highly Sexual | CG | #9_post_P25 | AACACAA | aci_P25 | post0_1 | 3283534 | 3129149 | 9231387 | 3077129 |
|  |  | #12_post_P26 | AACGTGA | aci_P26 | post0_2 | 3278966 | 3132026 |  |  |
|  |  | #12_post_P27 | AAGGAAT | aci_P27 | post0_3 | 3043110 | 2970212 |  |  |
|  | CHG | #9_post_P1 | GCTACAA | pstI_P1 | post0_1 | 2355786 | 2126705 | 6902709 | 2300903 |
|  |  | #12_post_P2 | AGCATCA | pstI_P2 | post0_2 | 3010022 | 2357298 |  |  |
|  |  | #12_post_P3 | CCATCCA | pstI_P3 | post0_3 | 3086079 | 2418706 |  |  |
|  | CHH | #9_post_P31 | CACGCTA | eco_P31 | post0_1 | 3136075 | 2447104 | 9040576 | 3013525 |
|  |  | #12_post_P32 | CAGGGCA | eco_P32 | post0_2 | 4345875 | 3607077 |  |  |
|  |  | #12_post_P33 | CAGGTGT | eco_P33 | post0_3 | 3485928 | 2986395 |  |  |
| **Total Postmeiosis**  **Highly Sexual** |  |  |  |  |  | **29025375** | **25174672** |  | **2797185** |
| Postmeiosis  Highly Aposporic | CG | #15_post_P25 | AACACAA | aci_P25 | post25_1 | 4894638 | 4764689 | 11746109 | 3915370 |
|  |  | #15_post_P26 | AACGTGA | aci_P26 | post25_2 | 3315593 | 3209057 |  |  |
|  |  | #39_post_P27 | AAGGAAT | aci_P27 | post25_3 | 3835936 | 3772363 |  |  |
|  | CHG | #15_post_P1 | GCTACAA | pstI_P1 | post25_1 | 2776134 | 2662589 | 8511755 | 2837252 |
|  |  | #15_post_P2 | AGCATCA | pstI_P2 | post25_2 | 3210029 | 2877491 |  |  |
|  |  | #39_post_P3 | CCATCCA | pstI_P3 | post25_3 | 3204307 | 2971675 |  |  |
|  | CHH | #15_post_P31 | CACGCTA | eco_P31 | post25_1 | 4033918 | 3376148 | 14289691 | 4763230 |
|  |  | #15_post_P32 | CAGGGCA | eco_P32 | post25_2 | 5074325 | 4404007 |  |  |
|  |  | #39_post_P33 | CAGGTGT | eco_P33 | post25_3 | 7115792 | 6509536 |  |  |
| **Total Postmeiosis**  **Highly Aposporic** |  |  |  |  |  | **37460672** | **34547555** |  | **3838617** |
| **Total** |  |  |  |  |  | **151740860** | **133906148** |  | **3719615** |

.

**Table S6**: Mapping of DMCs from premeiosis and postmeiosis on to the *Setaria italica* genome.

|  |  | | **Total number of DMCs by *Setaria* *italica* Chromosome (Relative number)^1^** | | | | | | | | |  |
| --- | --- | --- | --- | --- | --- | --- | --- | --- | --- | --- | --- | --- |
|  | **Context** | | **Si1** | **Si2** | **Si3** | **Si4** | **Si5** | **Si6** | **Si7** | **Si8** | **Si9** |  |
| Premeiosis | CG | | 6 (0.14) | 3 (0.06) | 5 (0.1) | 1 (0.02) | 12 (0.25) | 2 (0.06) | 5 (0.14) | 2 (0.05) | 9 (0.15) |  |
|  | CHG | | 27 (0.64) | 25 (0.51) | 28 (0.55) | 11 (0.27) | 35 (0.74) | 14 (0.39) | 21 (0.58) | 4 (0.1) | 28 (0.47) |  |
|  | CHH | | 317 (7.52) | 173 (3.52) | 302 (5.96) | 143 (3.54) | 349 (7.39) | 121 (3.36) | 158 (4.39) | 110 (2.7) | 243 (4.12) |  |
|  | | **Total Pm** | **350 (8.3)** | **201 (4.09)** | **335 (6.61)** | **155 (3.84)** | **396 (8.38)** | **137 (3.8)** | **184 (5.12)** | **116 (2.85)** | **280 (4.75)** | **2,154 (12.1)** |
| Postmeiosis | CG | | 262 (6.22) | 172 (3.5) | 253 (4.99) | 134 (3.32) | 318 (6.73) | 86 (2.39) | 161 (4.48) | 70 (1.72) | 278 (4.71) |  |
|  | CHG | | 543 (12.88) | 448 (9.11) | 587 (11.59) | 258 (6.38) | 637 (13.48) | 238 (6.61) | 358 (9.95) | 152 (3.74) | 619 (10.5) |  |
|  | CHH | | 52 (1.23) | 22 (0.45) | 36 (0.71) | 23 (0.57) | 41 (0.87) | 15 (0.42) | 25 (0.7) | 13 (0.32) | 35 (0.59) |  |
|  | | **Total Po** | **857 (20.33)** | **642 (13.05)** | **876 (17.29)** | **415 (10.27)** | **996 (21.08)** | **339 (9.41)** | **544 (15.13)** | **235 (5.78)** | **932 (15.8)** | **5,836 (18.4)** |

^1^ Relative number is obtained by the total number of DMCs per chromosome size (Mpb); Pm: premeiosis, Po: postmeiosis

**Table S7**: Sample description. The MCSeEd technique was applied to two biological and one technical replicates for both sexual and aposporic samples (0 and 25) in two developmental stages (premeiosis and postmeiosi). Each replicate was independently digested with context-specific (AciI/CG, PstI/CHG, EcoT22I/CHH) sensitive methylation enzymes in combination with the nonsensitive MseI and were identified by a specific code (P1, P2, P3, P25, P26, P27, P31, P32, P33). Then, libraries were pooled by stage and reproductive development, and each library was distinguished from each other by using the specific Illumina Indexes.

| Stage | Reproduction | Sample | Plant | *Pst* I  (CHG) | *Aci*I  (CG) | *Eco*T22I  (CHH) | Illumina Index |
| --- | --- | --- | --- | --- | --- | --- | --- |
| Premeiosis | Highly Sexual | pre0_1 | F_1_#9 | P1 | P25 | P31 | 12 (CTTGTA) |
|  |  | pre0_2 | F_1_#9 | P2 | P26 | P32 |  |
|  |  | pre0_3 | F_1_#12 | P3 | P27 | P33 |  |
|  | Highly Aposporic | pre25_1 | F_1_#15 | P1 | P25 | P31 | 15 (ATGTCA) |
|  |  | pre25_2 | F_1_#39 | P2 | P26 | P32 |  |
|  |  | pre25_3 | F_1_#39 | P3 | P27 | P33 |  |
| Postmeiosis | Highly Sexual | post0_1 | F_1_#9 | P1 | P25 | P31 | 14 (AGTTCC) |
|  |  | post0_2 | F_1_#12 | P2 | P26 | P32 |  |
|  |  | post0_3 | F_1_#12 | P3 | P27 | P33 |  |
|  | Highly Aposporic | post25_1 | F_1_#15 | P1 | P25 | P31 | 16 (CCGTCC) |
|  |  | post25_2 | F_1_#15 | P2 | P26 | P32 |  |
|  |  | post25_3 | F_1_#39 | P3 | P27 | P33 |  |

**Table S8:** Enzyme Specificity

| Enzyme | Recognition/cutting Site | Methylation Sensitivity | Blocked Site | Methylation Context* |
| --- | --- | --- | --- | --- |
| *Aci*I | C'CGC / G'CGG | yes | C^5m^CGC / G^5m^CGG | CG |
| *Pst*I | CTGCA'G | yes | CTG^5m^CAG | CHG |
| *EcoT22*I | ATGCA'T | yes | ATG^5m^CAT | CHH |
| *Mse*I | T'TAA | No |  | No |

*H: A, C o T
