# Supplementary figures and images for: Differential Epigenetic Marks Are Associated with Apospory Expressivity in Diploid Hybrids of Paspalum rufum
Source: Plants (Basel). 2021 Apr 17;10(4):793. doi: 10.3390/plants10040793 (PMC8072704; doi:10.3390/plants10040793)

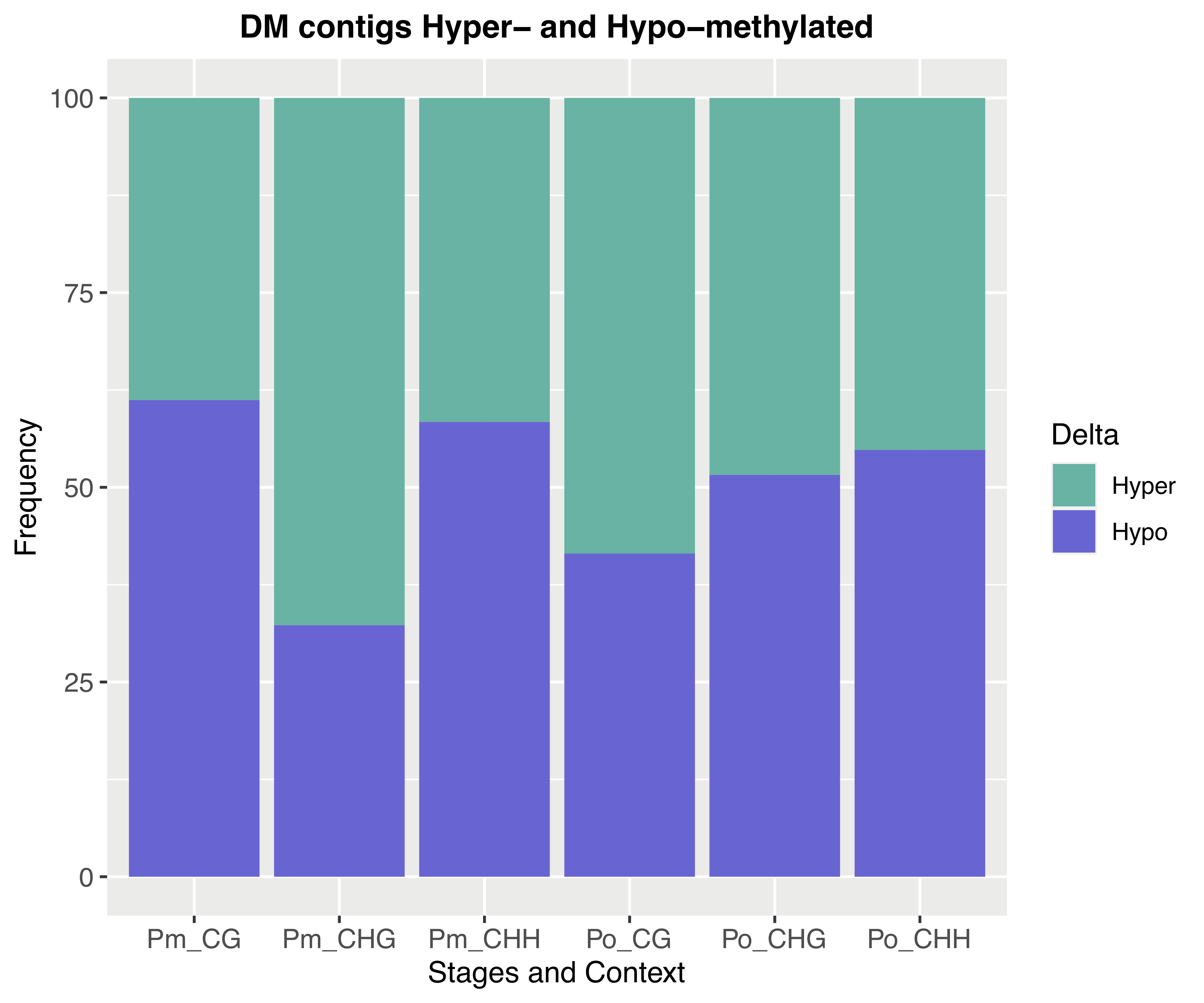

Supplement: Supplementary file 1 [file plants-10-00793-s001.zip › FigureS1.tiff]
